# Impaired Color Recognition in *HCN1* Epilepsy: A Single Case Report

**DOI:** 10.3389/fneur.2022.834252

**Published:** 2022-03-10

**Authors:** Chaseley E. Mckenzie, Chen-Jui Ho, Ian C. Forster, Ming S. Soh, A. Marie Phillips, Ying-Chao Chang, Ingrid E. Scheffer, Christopher A. Reid, Meng-Han Tsai

**Affiliations:** ^1^Early Development Division, Florey Institute of Neuroscience and Mental Health, Parkville, VIC, Australia; ^2^Division of Epilepsy, Department of Neurology, Kaohsiung Chang Gung Memorial Hospital, Kaohsiung, Taiwan; ^3^School of Biosciences, The University of Melbourne, Parkville, VIC, Australia; ^4^Department of Pediatrics, Kaohsiung Chang Gung Memorial Hospital, Kaohsiung, Taiwan; ^5^Epilepsy Research Centre, Department of Medicine, University of Melbourne, Austin Health, Heidelberg, VIC, Australia; ^6^Department of Paediatrics, Murdoch Children's Research Institute, Royal Children's Hospital, University of Melbourne, Parkville, VIC, Australia; ^7^School of Medicine, College of Medicine, Chang Gung University, Taoyuan, Taiwan; ^8^Genomics and Proteomics Core Laboratory, Department of Medical Research, Kaohsiung Chang Gung Memorial Hospital, Kaohsiung, Taiwan

**Keywords:** *HCN1*, epilepsy, developmental and epileptic encephalopathy, color vision, missense

## Abstract

Variants in *HCN1* are associated with a range of epilepsy syndromes including developmental and epileptic encephalopathies. Here we describe a child harboring a novel *de novo HCN1* variant, E246A, in a child with epilepsy and mild developmental delay. By parental report, the child had difficulty in discriminating between colors implicating a visual deficit. This interesting observation may relate to the high expression of HCN1 channels in rod and cone photoreceptors where they play an integral role in shaping the light response. Functional analysis of the *HCN1* E246A variant revealed a right shift in the voltage dependence of activation and slowing of the rates of activation and deactivation. The changes in the biophysical properties are consistent with a gain-of-function supporting the role of *HCN1* E246A in disease causation. This case suggests that visual function, including color discrimination, should be carefully monitored in patients with diseases due to *HCN1* pathogenic variants.

## Introduction

Pathogenic hyperpolarization-activated cyclic nucleotide-gated channel 1 (*HCN1*) variants are strongly associated with genetic epilepsies (1–4). Epilepsies caused by *de novo HCN1* variants tend to be the severe developmental and epileptic encephalopathies (DEEs), while inherited variants cause milder epilepsy syndromes such as genetic epilepsy with febrile seizures plus (GEFS+) (2, 3). Although much focus has been on the epileptology and developmental delay of *HCN1* disease, there may be pathology in other organs in which HCN1 channels play a role. One such organ is the retina in which HCN1 channels are highly expressed in the inner segment of the photoreceptor of rod and cone cells (5, 6). Here we present a patient harboring a novel *de novo HCN1* E246A variant with mild epilepsy and developmental delay. The patient has difficulty in identifying colors suggesting a visual deficit. Here we aimed to assess the biophysical properties of the *HCN1* E246A variant to see if dysfunction of this gene could underlie both the epilepsy and the unusual clinical finding of poor color discrimination.

## Methods

### Proband

The patient was recruited through the epilepsy genetics research program at Kaohsiung Chang Gung Memorial Hospital, Taiwan and the local human research committee approved the study. Written informed consent from the patient's parents was obtained. Whole exome sequencing (WES) and Sanger validation were performed as previously described (7).

Site-directed mutagenesis and cRNA preparation: cDNA encoding wild-type (WT) human HCN1 (RefSeq NM_021072.4, Ensemble database) was subcloned into the pGEMHE-MCS vector as previously described (8). The E246A variant was introduced by site-directed mutagenesis and the plasmid sequence was verified by Sanger sequencing, then linearized with NheI-HF (New England Biolabs) and purified using QIAquick PCR Purification Kit (QIAGEN). The concentration and quality of the linearized plasmid were confirmed using NanoDrop Spectrophotometer (Thermo Fisher Scientific) and gel electrophoresis. Linearized plasmid was transcribed to cRNA using the T7 mMessage mMachine kit (Ambion) and purified using RNeasy Mini Kit (QIAGEN). NanoDrop Spectrophotometer and gel electrophoresis were used to check the concentration and quality of cRNA. cRNAs were stored at −80°C.

### Oocyte Electrophysiology

Oocytes were prepared from adult female *Xenopus laevis* frogs as previously described (9). To express different ratios of protein, cRNA of the same concentration (200 ng/μl) was injected to a total of 50 nL volume. WT alone, E246A and WT + E246A (premixed) were injected to model WT, “homozygous mutant” and “heterozygous” conditions, respectively. Injected oocytes were maintained in ND96 storage solution (in mM: 96 NaCl, 2 KCl, 1 MgCl26H2O, 1.8 CaCl22H2O, 5 HEPES, 50 mg/L of gentamicin, pH 7.4) at 17°C for 2–3 days before experimentation. For electrophysiological recordings oocytes were superfused with a solution that contained (in mM) 100 KCl, 1.8 BaCl2, 1 MgCl2, 10 HEPES, pH 7.4 adjusted with TRIS (100 K solution). Control (non-injected) oocytes from the batch were checked to ensure that there was low endogenous leak.

Standard two-electrode voltage clamp hardware was used (TEC-05X or TEC10X, NPI), with series resistance (Rs) compensation applied. Electrodes were filled with 3M KCl and had resistances between 0.5 and 1.5 MΩ. Voltage clamp control and data acquisition were under software control (pCLAMP version 8–10, Molecular Devices). All experiments were performed at 18–20°C. Current-voltage (*I–V*) curves were generated using an activation voltage step protocol with 10 mV steps of 2.5 s duration from a −30 mV holding potential to test potentials in the range −120 mV to +20 mV. Steady-state current was measured over a 200 ms interval at the end of the test pulse. To characterize the rate of activation, we fitted a single exponential function to the data, commencing after the initial inflection (10). We found that a two exponential fit described the time course better for more hyperpolarized potentials, however the fit became less reliable for potentials more positive than −50 mV and therefore we report here the single exponential fit data only. For determining the deactivation kinetics, a deactivation protocol was used in which the oocyte was first voltage clamped to −120 mV for 2.5 s to open all channels, and the membrane voltage was then stepped to test potentials in the range −110 to +40 mV. The channel closure time course was well-described by a single exponential function over the whole test voltage range when taking account of the initial inflection for hyperpolarized potentials. When exponential fits were poor for a given data set that oocyte was removed for the purpose of that specific analysis. For determining the instantaneous tail currents, the activation data were first baseline-corrected at the end of recording; the instantaneous current at each test potential was estimated shortly after the capacitive transient had settled and the data for each cell fit with a form of the Boltzmann function to estimate the maximum current span. The data were normalized to the estimated maximum tail current and pooled. A Boltzmann fit of these data yielded estimates of the midpoint voltage (*V*_0.5_) and apparent valence (*z*) (9). Data were sampled at 200 μs/point and low pass filtered at 500 Hz. Curve fitting of the activation and deactivation kinetics was performed using Clampfit routines.

### Statistical Analysis

Data points in graphs are shown as mean ± sd. Data sets were compared to wild-type using a one-way ANOVA (Graphpad, Prism) with Dunnett's *post-hoc* test.

## Results

### Patient's Phenotype

The proband was a 6-year-old boy who presented with focal clonic seizures at age 7 months after a hot bath. EEG showed left frontal epileptiform activity. Brain MRI was normal. He was started on phenytoin which was not effective but, subsequently, seizures were controlled with sodium valproate. Mild attention deficit hyperactivity disorder was diagnosed at 2.5 years old and he received early intervention. At 4.3 years, he had mild global developmental delay. When he was 6 years old, his parents observed him using strange colors to draw fruits and he had difficulties using colors with a similar hue. They also observed that he had difficulty in categorizing colors such as yellow, blue, and green, but he could match objects that had the exact same color.

### Genetic Study

**Figure 1:**
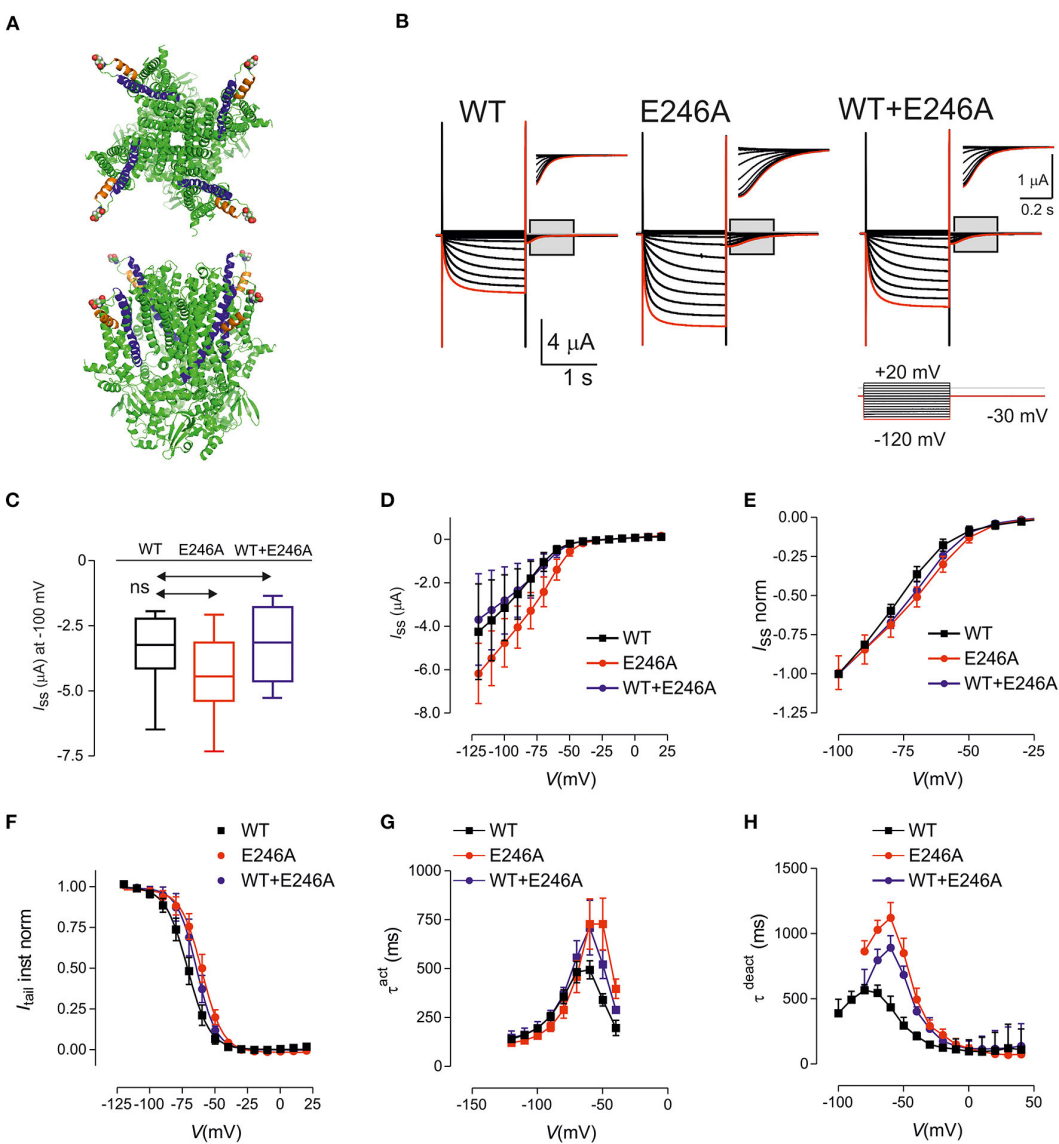
HCN1 E246A variant causes changes in the biophysical properties consistent with gain of function. **(A)** Structure of human HCN1 showing location of E246 (shown as spheres). Based on PDB 5u60 for human HCN1 in the depolarized (closed) conformation, rendered using PyMOL (The PyMOL Molecular Graphics System, Version 2.3.4 Schrodinger, LLC.). Upper panel: view from external medium; lower panel: side view. **(B)** Representative voltage clamp data from oocytes expressing HCN1 wild-type (WT) (left); E246A (middle) and co-expressed WT + E246A (right). Each dataset shows current traces in response to a series of voltage steps (inset) from the holding potential (−30 mV) to test potentials in the range −120 to + 20 mV (activation protocol, see Methods). Gray boxed areas have been enlarged to show tail currents. Note the different scales. Red trace corresponds to voltage step at −120 mV. **(C)** Box and Whisker plot of maximal steady-state current for HCN1 WT (*n* = 9); E246A (*n* = 9),and WT + E246A (*n* = 9) measured at end of test pulse to −100 mV; ns, not significant. **(D)** Pooled current-voltage (I–V) data shows a shift in the voltage-dependence of steady state activation for “homozygous” (*n* = 9 each group) compared to WT (*n* = 9). **(E)** Normalized, pooled I-V data reveals the right shift in steady-state activation; each data set was normalized to the steady-state current at −100 mV. The “heterozygous” data lies between the “homozygous” and WT data sets (*n* = 9 for each data set). **(F)** Normalized, instantaneous tail currents fit with a single Boltzmann function. Fit values were: *V*_0.5_ −71.1 ± 0.4 mV (WT); −60.1 ± 0.4 mV (E246A); −64.1 ± 0.4 mV (WT+E246A) and z: 2.92 ± 0.11 (WT); 3.09 ± 0.12 (E246A); 3.34 ± 0.15 (WT+E246A). **(G)** Mean activation time constant τ ^act^ obtained by fitting the time-dependent component of activating current with a single exponential function (see Methods). **(H)** Mean deactivation time constant obtained by fitting the time-dependent component of deactivating current with a single exponential function. Data points were joined for visualization. Data are expressed as mean ± s.d; statistical comparisons were made to wild-type using one-way ANOVA with Dunnett's *post-hoc* test (see Supplementary Tables 1–3 for detailed analyses).

We performed whole exome sequencing (WES) on the proband and identified a novel heterozygous *HCN1* missense variant (NM_021072) in exon 2 of *HCN1*: c.A737C, p.Glu246Ala (E246A). E246 is in the linker region between S3 and S4 of *HCN1* (Figure 1A), which is located in the Pathogenic Enriched Region (PER) based on the PER viewer (11). Sanger sequencing of the parent-child trio showed it had arisen *de novo*. The variant was not presented in control databases, such as ExAC, gnomAD and TaiwanBiobank, or ClinVar. Multiple *in-silico* algorithms predict the variant to be deleterious with a CADD score of 25.9. The variant was classified as “likely pathogenic” based on American College of Medical Genetics and Genomics Guidelines: PS2 + PM1 + PM2 + PP3 (12).

### Functional Characterization

The functional consequences of the *HCN1* E246A variant were investigated using two-electrode voltage-clamp recordings in *Xenopus laevis* oocytes (Figure 1B). The variant was expressed and characterized under “homozygous” and “heterozygous” (co-expressed, modeling the patient situation) conditions. Typical traces of HCN currents recorded from cells from the same donor frog expressing WT, “homozygous” E246A channels or “heterozygous” WT+E246A channels are shown in Figure 1B. Pooled currents, measured at −100mV, showed similar functional expression (Figures 1C,D; Supplementary Table 1; *p* = 0.42, *F* ratio = 0.91, DF*n* = 2, DFd = 24, *n* = 9 total, 3 oocytes each from three different donor frogs). Normalized steady state current-voltage relations revealed a small shift in the voltage-dependence of steady state activation for E246A compared to WT (Figure 1E). Consistent with this, instantaneous tail currents showed a right shift in voltage-dependence, which indicated an increased probability of channels opening at more depolarized potentials (Figure 1F). Boltzmann fits to the tail currents revealed a significant difference in *V*_0.5_ (−71.1±0.4 mV (WT); −60.1 ± 0.4 mV (E246A); −64.1 ± 0.4 mV (WT+E246A), p < 0.05 for each comparison, *F* ratio = 230.3, DF*n* = 2, DFd = 24, see Supplementary Table 1), while the apparent valence was unchanged (2.92 ± 0.11 (WT); 3.09 ± 0.12 (E246A); 3.34 ± 0.15 (WT+E246A), *n* = 9 *p* = 0.09 for each comparison, *F* ratio = 2.68, DF*n* = 2, DFd = 24). At more depolarised potentials, the rate of activation of both “homozygous” and “heterozygous” channels were significantly slowed compared with WT (Figure 1G, see Supplementary Table 2 for detailed statistical comparison). Finally, there was also a significant slowing of the deactivation rate of “homozygous” and “heterozygous” channels relative to WT at more hyperpolarised potentials, whereas the rates for all three constructs converged at depolarizing potentials (Figure 1H, see Supplementary Table 3 for detailed statistical comparison). Taken together, the HCN1 E246A variant caused a right shift of the voltage dependence of activation, as well as slowing the rates of both activation and deactivation.

## Discussion

Here we describe the novel *HCN1* E246A pathogenic variant, associated with well controlled focal epilepsy, mild developmental delay and parental report of visual dysfunction, that results in a net gain of channel function. This is due to a right-shift in the voltage of activation as well as a slowing of the deactivation kinetics. An increase in the number of HCN1 channels opening at around resting membrane potentials results in a depolarisation of central neurons (9). As a consequence, neurons would be expected to fire more readily, increasing excitability that presumably leads to seizures (9, 13).

Gain of function, through a variety of biophysical mechanisms that include hyperpolarising shifts in V_0.5_ and loss of voltage sensitivity, is common in *HCN1* epilepsy (2–4). Our functional studies have established that the E → A substitution at position 246 in the external linker between S3 and S4 of the HCN1 voltage sensing domain significantly effects voltage-dependent gating of HCN1 channels, which also further changed the ACMG classification from “likely pathogenic” to “pathogenic” (PS2 + PS3 + PM1 + PM2 + PP3). Similarly, the HCN1 M243R variant found in a patient with DEE marginally shifts voltage dependence of activation with significant changes in the slope factor further implicating a role of this linker region in voltage sensitivity (2). While the details of the S4 helix movement during HCN1 channel activation, leading to pore opening are now becoming clearer (14), the role of residues in the S3-S4 linker will require further clarification. Findings in *HCN1* DEE reported here and by others will serve as a starting point for more detailed biophysical analysis.

It must be noted that the oocyte functional analysis assay is opaque to many aspects through which *HCN1* variants may cause disease. Neurons have a complexity that include a milieu of proteins that could influence a variant-mediated change in function including second messenger cascades (eg cAMP) or accessory subunits that influence trafficking (e.g., Trip8) (15). Furthermore, oocytes do not have the structural complexity seen in neurons, especially important for HCN1 which is distributed non-uniformly across different neuronal compartments (15). Temperature is another potential confounder given that oocytes can only be recorded reliably at relatively low temperatures. Nevertheless, oocytes remain a useful model to interrogate biophysical changes in the HCN1 protein. For example, we recently reported brain slice recording of I_h_ from a knock-in mouse model of *HCN1* DEE that recapitulated the “cation leak” phenotype observed in oocytes (9). Additional experimentation in model systems are required to better explore the relationship between *HCN1* pathogenic variants and changes in the visual system.

Interestingly, patients treated with ivabradine, a broad-spectrum HCN blocker, have reported the appearance of bright areas in the visual field (phosphenes) which are often triggered by changes in luminosity from dark to light (16). Ivabradine is not brain penetrant and blocks HCN1 channels in mouse rod photoreceptors implicating changes in retina function as the cause of phosphenes (17). HCN1 channels are specifically strongly enriched in rod and cone photoreceptor inner segments where they play a critical temporal role in shaping the light response (5, 6). In the dark, photoreceptors remain unstimulated and depolarised. Upon illumination, rods and cones are stimulated and become hyperpolarised. HCN1 channels become activated during this membrane hyperpolarisation in bright light and depolarise the cell toward the dark membrane potential, making the light response more transient (18). It should be noted that HCN1 channels also play a critical role in the visual cortex where they are important modulators of resting membrane potential and synaptic integration in pyramidal neurons (15). It is plausible that cortical dysfunction could also cause or contribute to the visual deficits reported here.

Visual deficits have not previously been reported in *HCN1* epilepsies. The basis of this is unclear but may relate to the severe nature of most HCN1 DEEs (2, 3). Whether visual problems are present in mild *HCN1-*GEFS+ patients also remain to be determined. We specifically report a deficit in color discrimination. However, given the critical role of HCN1 channels in returning potentials toward rest, other retinal functions are likely to be affected. These include reduced capacity to see fine detail, which may require more contrast to enhance visibility, and changes in the ability to detect motion. Further testing of these deficits in patients with *HCN1* epilepsies is warranted but will be difficult in patients with DEEs who have limited ability to communicate.

Gain-of-function *HCN1* DEE has an emerging pharmacological profile. Case reports suggest that phenytoin and lamotrigine, antiseizure drugs that predominantly act through voltage-dependent sodium channel block, worsen seizures in patients harboring *HCN1* gain-of-function variants (2, 9, 13). In contrast, sodium valproate may be effective in partially controlling seizures (9). This pharmacological profile is mirrored in three mouse models that have been engineered to carry HCN1 gain-of-function variants (9, 13). Interestingly, our patient with the *HCN1* E246A variant did not respond to phenytoin but was controlled with valproate monotherapy.

In summary, we describe a novel gain-of-function *HCN1* variant in a DEE patient. A visual deficit was observed consistent with an important role of HCN1 channels in retinal function. Where possible, visual function should be tested in all patients harboring *HCN1* pathogenic variants as correcting or adapting to any dysfunction may greatly improve the quality of life of the patient.

## Data Availability Statement

The datasets presented in this article are not readily available due to ethical and privacy restrictions. Requests to access the datasets should be directed to the corresponding author.

## Ethics Statement

The studies involving human participants were reviewed and approved by Kaohsiung Chang Gung Memorial Hospital. Written informed consent to participate in this study was provided by the participants' legal guardian/next of kin.

## Author Contributions

All authors listed have made a substantial, direct, and intellectual contribution to the work and approved it for publication.

## Funding

This research was funded by CMRPG8K1441 to M-HT from Kaohsiung Chang Gung Memorial Hospital, Taiwan; MOST-110-2314-B-182A-076-MY3 to M-HT from the Ministry of Science and Technology, Taiwan; and NHRI-EX110-11022NI to M-HT from National Health Research Institute, Taiwan. This study was supported by an Australian National Health and Medical Research Council (NHMRC) Program Grant (1091593) to IS and CR and Senior Investigator Grant (1172897) to IS.

## Conflict of Interest

The authors declare that the research was conducted in the absence of any commercial or financial relationships that could be construed as a potential conflict of interest.

## Publisher's Note

All claims expressed in this article are solely those of the authors and do not necessarily represent those of their affiliated organizations, or those of the publisher, the editors and the reviewers. Any product that may be evaluated in this article, or claim that may be made by its manufacturer, is not guaranteed or endorsed by the publisher.
