# Supplementary material for: Impaired Color Recognition in HCN1 Epilepsy: A Single Case Report
Source: Front Neurol. 2022 Mar 10;13:834252. doi: 10.3389/fneur.2022.834252 (PMC8960314; doi:10.3389/fneur.2022.834252)
Supplement: Supplementary file 1 [file Table_1.DOCX]

Supplementary table 1: Summary of electrophysiology parameters (expressed as mean ± s.d) of hHCN1 wild-type and variants.

|  | **Number of oocytes** | **I_ss_ (µA) at -100 mV** | **V_1/2_ (mV)** | ***z* valence** |
| --- | --- | --- | --- | --- |
| Wild-type | 9 | -3.4 ± 1.3 | -71.1 ± 0.4 | 2.92 ± 0.11 |
| E246A | 9 | -4.4 ± 1.5 | -60.1 ± 0.4**** | 3.09 ± 0.12 |
| WT+E246A | 9 | -3.2 ± 1.5 | -64.1 ± 0.4**** | 3.34 ± 0.15 |

**** P < 0.0001 from one-way ANOVA with Dunnett’s post-hoc, compared to WT.

Supplementary table 2: Statistical comparison of tau activation (in milliseconds, expressed as mean ± s.d). Numbers in brackets represent F-ratio, DFn and DFd respectively.

| Voltage | Wild-type (n=9) | E246A (n=9) | WT+E246A (n=5) |
| --- | --- | --- | --- |
| **-40 mV** (55.9, 2, 20) | 196.9 ± 38.9 | 397.0 ± 49.4**** | 289.7 ± 11.5** |
| **-50 mV** (40.18, 2, 20) | 340.5 ± 31.5 | 728.1 ± 132.1**** | 521.8 ± 72.2** |
| **-60 mV** (12.52, 2, 20) | 494.1 ± 46.5 | 728.1 ± 128.5*** | 709.8 ± 139.4** |

** P < 0.01, *** P < 0.001, **** P < 0.0001 from one-way ANOVA with Dunnett’s post-hoc, compared to WT.

Supplementary table 3: Statistical comparison of tau deactivation (in milliseconds, expressed as mean ± s.d). Numbers in brackets represent F-ratio, DFn and DFd respectively.

| Voltage | Wild-type (n=9) | E246A (n=8) | WT+E246A (n=8) |
| --- | --- | --- | --- |
| **-80 mV** (21.6, 2, 22) | 565.4 ± 43.5 | 864.2 ± 81.27**** | 578.9 ± 147.3 |
| **-70 mV** (96.5, 2, 22) | 544.9 ± 59.8 | 1030 ± 72.7**** | 791.1 ± 82.9**** |
| **-60 mV** (108.2, 2, 22) | 435.4 ± 84.2 | 1121 ± 117.8**** | 892.5 ± 91.4**** |
| **-50 mV** (100.7, 2, 22) | 295.7 ± 61.6 | 850.7 ± 114.1**** | 684.4 ± 66.2**** |
| **-40 mV** (56.7, 2, 22) | 212.6 ± 30.9 | 494.5 ± 87.1**** | 403.7 ± 33.9**** |
| **-30 mV** (16.4, 2, 22) | 149.7 ± 19.8 | 291.8 ± 46.7**** | 270.8 ± 84.3*** |
| **-20 mV** (18.8, 2, 22) | 126.0 ± 19.5 | 222.3 ± 44.9**** | 175.9 ± 28.8** |

** P < 0.01, *** P < 0.001, **** P < 0.0001 from one-way ANOVA with Dunnett’s post-hoc, compared to WT.
