# Supplementary figures and images for: Impaired Color Recognition in HCN1 Epilepsy: A Single Case Report
Source: Front Neurol. 2022 Mar 10;13:834252. doi: 10.3389/fneur.2022.834252 (PMC8960314; doi:10.3389/fneur.2022.834252)

A

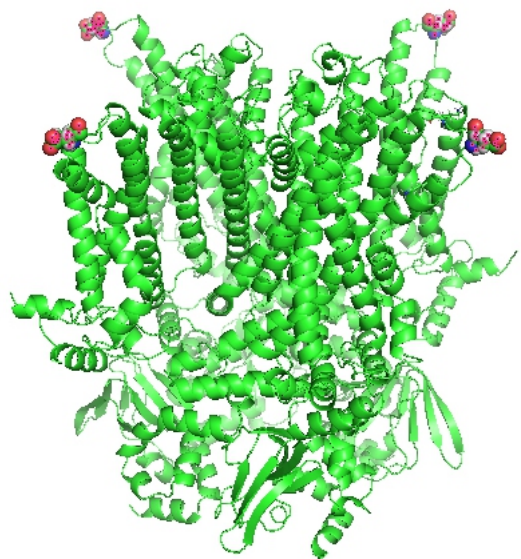

B

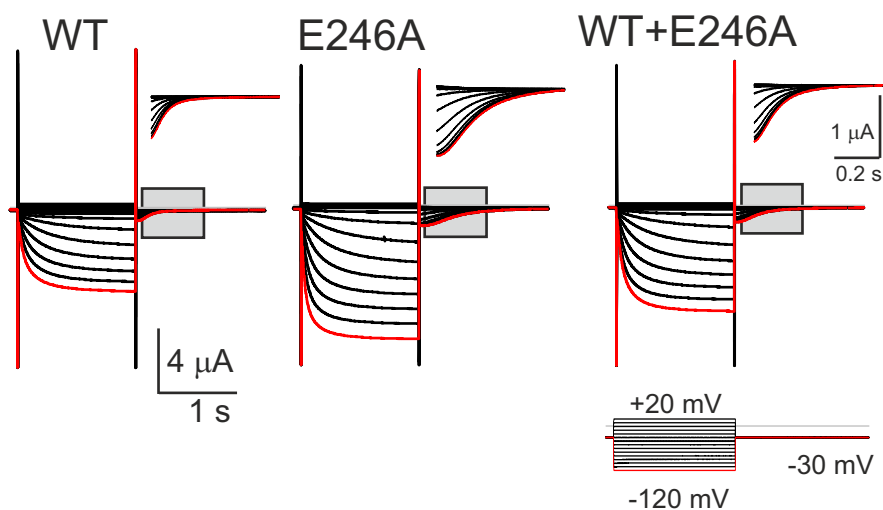

C

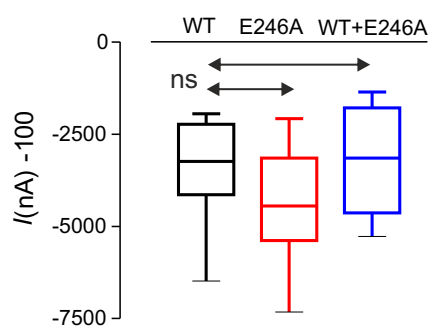

D

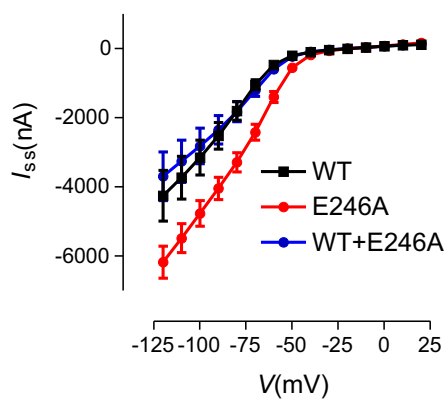

E

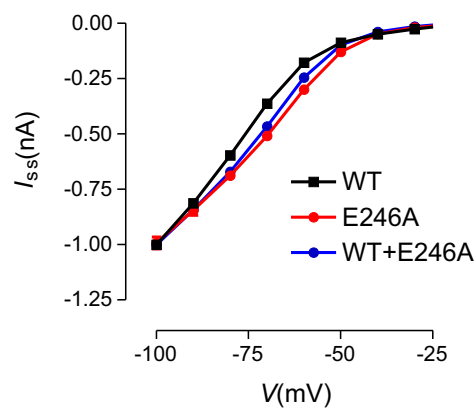

F

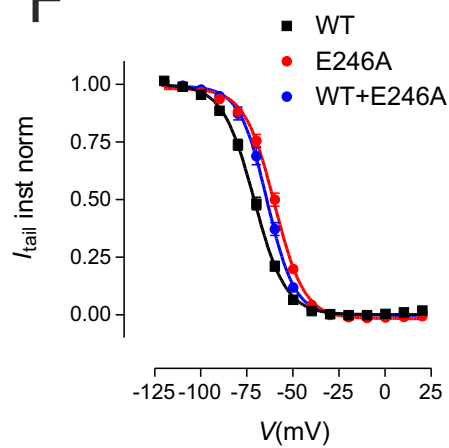

G

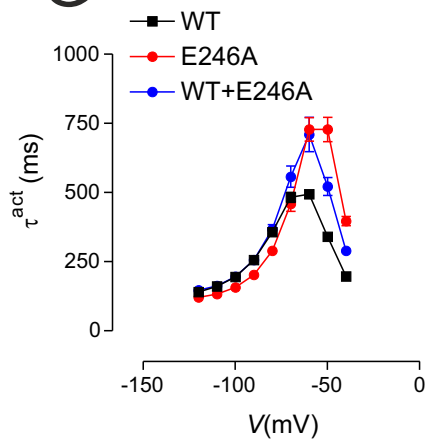

H

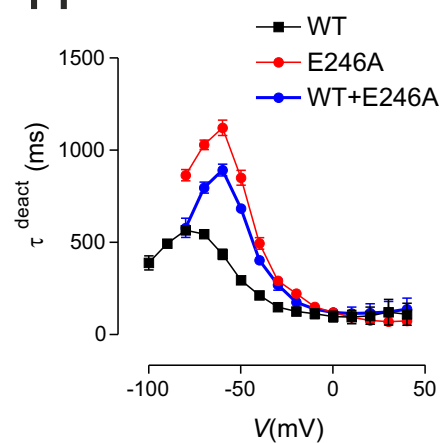

Supplement: Supplementary file 2 [file Data_Sheet_1.PDF]
